# Saddle Pulmonary Embolism in Patients with Cancer in the Era of Incidental Events: Clinical Findings and Outcomes in a Single Centre Cohort

**DOI:** 10.1055/a-1897-7061

**Published:** 2022-12-14

**Authors:** Mario Aramberri, Mariana Benegas, Marcelo Sanchez, Diego Muñoz-Guglielmetti, Carles Zamora, Adrián García-Villa, Carmen Diaz-Pedroche, Carme Font

**Affiliations:** 1 Department of Internal Medicine, Hospital de Galdakao-Usansolo, Galdakao, Spain; 2 Department of Radiology, Hospital Clinic de Barcelona, Barcelona, Spain; 3 Department of Radiation Oncology, Barcelona, Spain; 4 Department of Medical Oncology, Hospital Clinic de Barcelona, Barcelona, Spain; 5 Department of Internal Medicine, Hospital Nuestra Señora del Prado, Talavera de la Reina, Spain; 6 Department of Internal Medicine, Hospital Universitario 12 de Octubre, Madrid, Spain

**Keywords:** cancer, incidental findings, pulmonary embolism, thrombosis, venous thromboembolism

## Abstract

**Background** There is scarce information regarding the prevalence and clinical impact of saddle pulmonary embolism (PE) in patients with cancer.

**Objectives** This study aimed to assess the prevalence, clinical findings, and short-term outcomes of patients with cancer-related saddle PE including acute symptomatic and unsuspected events.

**Patients/Methods** Consecutive patients with cancer-related PE (March 1, 2006–October 31, 2014) were retrospectively reviewed by a chest radiologist to assess PE burden and signs of right ventricular (RV) overload. The clinical outcomes within 30 days were evaluated according to saddle versus nonsaddle PE.

**Results** Thirty-six (12%) out of 289 patients with newly diagnosed cancer-related PE presented with saddle PE. Saddle PE was found in 21 cases (58%) with acute symptomatic PE and the remaining 15 cases (42%) were found as unsuspected findings. Patients with saddle PE had more frequently experienced a previous thrombotic event (31 vs. 13%; *p* = 0.008), and it occurred more frequently as an acute symptomatic event (58 vs. 39%; *p* = 0.025) compared with those with nonsaddle PE. Signs of RV overload including RV/left ventricle ratio ≥1 (22 vs. 4%; *p* < 0.001) and interventricular septum displacement (53 vs. 20%; *p* < 0.001) were also more common in patients with saddle PE compared with nonsaddle PE. Overall, PE-related mortality, venous thromboembolism recurrence, and major bleeding within 30 days were found to be similar according to saddle versus nonsaddle PE.

**Conclusion** Saddle PE is not uncommon in patients with cancer-related PE including in those with unsuspected PE. Similar 30-day outcomes were found according to saddle versus nonsaddle PE in our cohort.

## Introduction

Cancer and its treatment increase the risk of developing venous thromboembolism (VTE) including deep vein thrombosis (DVT) and pulmonary embolism (PE). There has been a progressive rise in the incidence of cancer-associated VTE compared with a steady incidence of VTE in the general population 1 within the context of (1) global longer life expectancy in the general population leading to more cancer diagnosis, (2) recent advances in tumor treatment significantly prolonging the survival of patients with cancer, 2 and (3) technological advances improving computerized tomography (CT) equipment and the broader use of CT scans resulting in the diagnosis of incidental or unsuspected VTE during routine examinations. 3

In this regard, incidental VTE and, especially, unsuspected PE (UPE) currently represent up to half of the VTE events in patients with cancer. 4 5 6 7 8 9 UPE is defined as an unsuspected filling defect in the pulmonary arteries identified on CT imaging performed for another indication, such as a routine staging scan to assess cancer disease status. The diagnosis and treatment of UPE is challenging for radiologists and clinicians.

In a recent systematic review, the median prevalence of UPE was reported to be 3.36%, ranging widely according to the underlying primary tumor. 10 Several cumulative observational studies have suggested similar outcomes regarding mortality when comparing incidental versus symptomatic VTE events. 11 12 13 Thus, in the absence of further knowledge, the current clinical guidelines of international societies recommend treating UPE similar to acute symptomatic PE. 8 14 15 16

The clinical severity of patients with PE ranges widely from completely asymptomatic to potentially life-threatening events. Risk-stratification models for acute PE include variables related to hemodynamic instability, right ventricle (RV) overload, and cardiac biomarkers. 16 17 18 19 The thrombotic burden of PE is not included in these models, since data regarding their prognostic impact is controversial. 20

**Fig. 1:**
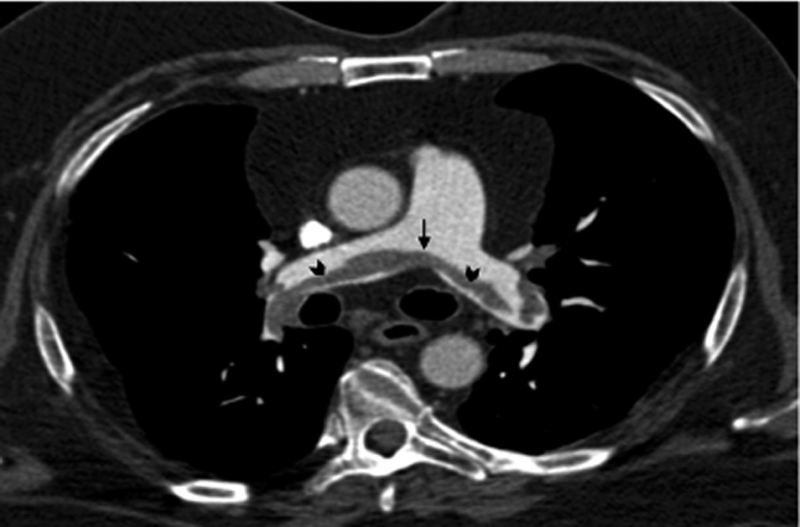
Chest CT angiography depicting a saddle PE. Image shows thrombus straddling the bifurcation of the pulmonary artery trunk (arrow) with extension into both main pulmonary arteries (arrowheads). CT, computed tomography; PE, pulmonary embolism.

In this regard, saddle PE represents a prime example of large thrombotic burden. The term “saddle PE” is a radiological concept commonly used in daily practice by clinicians and radiologists which is classically defined as a visible thrombus that straddles the bifurcation of the main pulmonary artery trunk, 21 22 as shown in Fig. 1 .

There is scarce information regarding the prevalence, clinical characteristics, and outcomes of patients with cancer and saddle PE apart from a few case reports in patients with suspected PE 23 24 and, more recently, in patients with UPE. 25 26

The aim of the present study was to assess the prevalence, clinical findings, and short-term outcomes of patients with cancer and saddle PE including acute symptomatic and unsuspected events.

## Methods

### Study Design and Setting

We performed a retrospective analysis of data prospectively collected from consecutive patients with cancer-associated PE from a local registry at the Medical Oncology Department in the Hospital Clinic Barcelona (Spain), an urban teaching hospital covering an area of 500,000 inhabitants as reported elsewhere. 5 27 28 29 Patients with radiologically confirmed PE with either acute symptomatic or unsuspected events between March 1, 2006, and October 31, 2014, were evaluated.

Eligible patients had been previously diagnosed with cancer, were >18 years of age and were diagnosed with cancer-associated PE by either CT angiography, specifically ordered to depict PE (acute suspected events), or a scheduled conventional CT scan (UPE). Patients diagnosed with lung perfusion scintigraphy were excluded from the present analysis. Patient management and follow-up was made according to the standard local clinical practice protocol as part of previous research and reported elsewhere. 27

The primary objective was to assess the prevalence, clinical characteristics, management, and short-term outcomes within 30 days of PE diagnosis in patients with saddle versus nonsaddle PE including patients with acute symptomatic and UPE events.

Secondary objectives included: (1) In-hospital outcomes, (2) radiological findings related to RV overload and additional radiological findings according to the presence of saddle versus nonsaddle PE, and (3) overall comparison of baseline characteristics and outcomes among patients with saddle PE according to the presence of acute symptomatic versus unsuspected events.

The study was approved by the local clinical research ethics board. Informed written consent was obtained from all the prospectively enrolled participants. Patient data were anonymized and deidentified prior to analysis.

## Demographic and Clinical Variables

The variables analyzed were collected from the hospital medical records and included demographic variables at PE diagnosis, as well as cancer-related variables, such as the location of primary tumor, the presence of metastases, anticancer treatment at PE diagnosis, and performance status according to the Eastern Cooperative Oncology Group scale.

Patients were considered to have an outpatient diagnosis of PE if the thrombotic event was detected during or before attendance to the emergency department (ED). Patients presenting an episode of VTE more than 7 days before PE diagnosis were classified having previous VTE, whereas those with a diagnosis of DVT within 7 days of PE diagnosis were considered to have concomitant DVT. Symptoms (dyspnea, chest pain, or hemoptysis) and vital signs (heart rate, systolic blood pressure, and oxygen saturation) were assessed on hospital arrival in patients diagnosed in the outpatient setting. In the case of hospital-acquired PE events, we collected the last vital signs registered before the diagnosis of PE. Causes of death were recorded according to a multiple-choice classification by the clinician in charge including pulmonary embolism, cancer progression, and/or other causes.

## Systematic Radiological Examination

CT scans were thoroughly reviewed by either one of two senior chest radiologists (M.B. and M.S.) as part of previously reported research. 5 27 28 29 Radiologist were blinded to clinical data and outcomes.

Conventional CT scans were performed with a dual CT scanner (Somatom scanner; Siemens Medical Solutions, Somatom Healthcare, Erlangen, Germany) using a 100-mL intravenous injection of nonionic contrast medium (300 mg/mL) infused at a rate of 3 mL/s. Conventional CT of the thorax was performed with an automatic detection (care bolus) of contrast in the ascending aorta, with 1.2-mm collimation and 5-mm reconstruction. Conventional CT of the abdomen and pelvis was performed from the diaphragm to the pubic symphysis 70 to 90 seconds after the infusion of the contrast medium for the chest CT.

The CT scan specific for the depiction of PE was performed with the 64 multidetector CT scan, with 0.6-mm collimation and 1-mm reconstruction including a CT pulmonary angiogram (CTPA) of the pulmonary arteries and lower limb venography. A thrombus in either scan was defined as a definite intraluminal filling defect identified on at least two consecutive transverse images. 30 31

Saddle PE is radiologically defined as “a thrombus that straddles the bifurcation of the pulmonary artery trunk, often with extension into both the right and left main pulmonary arteries.” 21 22

A thrombus was considered peripheral if its most proximal occluded artery was either a segmental or subsegmental artery. The presence of either a RV to left ventricle (LV) diameter ratio greater than or equal to 1, septum displacement, or contrast reflux to suprahepatic veins were considered signs of RV overload.

Analysis included other additional findings not directly related to PE such as the presence of lung nodules, cancer progression, carcinomatous lymphangitis, pleural effusion, pericardial effusion, and “other findings” (atelectasia, pneumonia, additional thrombus diagnosis, emphysema, pulmonary edema, pulmonary fibrosis, pneumothorax, ground-glass opacities, or chronic pulmonary embolism).

## Outcome Measures

The following 30-day outcome measures were included the following: (1) overall mortality, (2) PE-related mortality, (3) VTE recurrence defined as an objectively confirmed (with the Doppler ultrasound, CTPA, or lung perfusion scintigraphy) new episode of PE or DVT, and (4) major bleeding according to the International Society on Thrombosis and Haemostasis criteria (fatal bleed and/or symptomatic plus critical organ or reduction in hemoglobin >2 g/dL or transfusion of >2U of red blood cells). 32

All clinical data were reviewed and evaluated by the authors and collected in a specific database designed for the purpose of this study.

## Data Analysis

Descriptive statistics, including means, standard deviation, and percentages were used to summarize patient characteristics. Comparisons between saddle and nonsaddle PEs were made. The two-sided *t* -test was used to assess statistical significance for continuous variables and the Chi-square test or Fisher's exact test was used for categorical variables. A *p* < 0.05 was deemed to be statistically significant. The analyses were performed using SPSS v24 software.

## Results

**Fig. 2:**
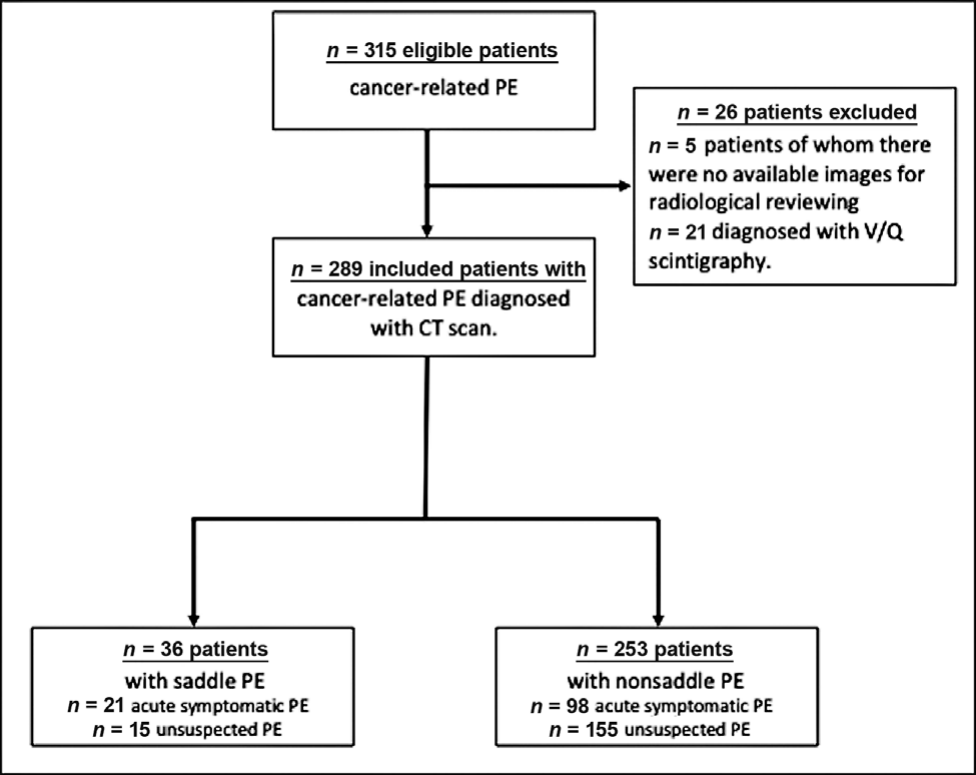
Flow chart of patient selection for the study. CT, computed tomography; PE, pulmonary embolism.

A total of 315 patients with cancer and PE were collected during the study period. Of these, 289 cases (39% female; mean age: 64 years) were diagnosed by CT scan with available imaging tests for radiological reviewing and were included in the study. No patient was lost to follow-up. Saddle PE was diagnosed in 36 of the 289 cases (12.5% of the overall cohort) including 21 (58%) acute symptomatic events and 15 (42%) cases of UPE as shown in the flowchart ( Fig. 2 ). Of note, saddle PE was found in 15 (8.8%) out of 170 patients with UPE.

### Saddle Pulmonary Embolism versus Nonsaddle Pulmonary Embolism

The main baseline characteristics and clinical features at presentation according to the presence of saddle versus nonsaddle PE are shown in Table 1 . No differences regarding gender, age, chronic lung or heart conditions, proportion of inpatients at PE diagnosis, and cancer type and anticancer therapies were observed on comparing patients with saddle versus nonsaddle PE. Of note, patients with saddle PE more frequently had a history of VTE than those with nonsaddle PE (31 and 13%, respectively; *p* = 0.008).

**Table 1:** Baseline characteristics according to the presence of saddle versus nonsaddle pulmonary embolism

	Overall ( *n* = 289) Mean ± SD/ *n* (%)	Saddle PE ( *n* = 36) Mean ± SD/ *n* (%)	Nonsaddle PE ( *n* = 253) Mean ± SD/ *n* (%)	*p* -Value
Age (y)	64 ± 11.5	65 ± 10.8	64 ± 11.8	>0.5
Gender (F)	112 (39)	14 (39)	98 (39)	>0.5
Chronic lung condition	62 (21)	7 (19)	55 (22)	>0.5
Chronic heart disease	18 (6)	1 (3)	17 (7)	>0.5
Inpatient at PE diagnosis	39 (13)	3 (8)	36 (14)	0.44
Previous VTE	45 (16)	11 (31)	34 (13)	**0.008**
Concomitant DVT	63 (22)	9 (25)	54 (21)	>0.5
Cancer type				0.45
Lung	99 (34)	10 (28)	89(35)	
Colorectal	40 (14)	7 (19)	33 (13)	
Genitourinary	39 (13)	4 (11)	35 (14)	
Gynecologic	33 (11)	5 (14)	28 (11)	
Upper GI	33 (11)	4 (11)	29 (11)	
Breast	15 (5)	2 (6)	13 (5)	
Other	30 (10)	4 (11)	26 (10)	
Metastatic cancer	236 (82)	27 (75)	209 (83)	0.27
Surgery	23 (8)	4 (11)	19 (8)	0.455
Chemotherapy	149 (52)	21 (58)	128 (51)	0.385
Radiological test				**0.025**
CT scan angiography (acute suspected PE)	119 (41)	21 (58)	98 (39)	
Conventional CT scan (UPE)	170 (59)	15 (42)	155 (61)	
PE symptoms				
None	134 (46)	11 (31)	123 (49)	**0.042**
Dyspnea	136 (47)	24 (67)	112 (45)	**0.005**
Chest pain	40 (14)	6 (17)	34 (13)	>0.5
Syncope	20 (7)	4 (11)	16 (6)	0.29
Hemoptysis	4 (1)	0 (0)	4 (2)	>0.5
Vital signs				
Arterial hypotension BP < 100 mm Hg	21 (7)	1 (3)	20 (8)	0.49
Tachycardia HR > 100 beats per minute	77 (27)	16 (44)	61 (24)	**0.01**
Oxygen saturation <90%	35 (12)	7 (19)	28 (11)	0.17
Oxygen saturation <95%	99 (34)	20 (56)	79 (31)	**0.004**

Abbreviations: BP, blood pressure; CT, computerized tomography; DVT, deep vein thrombosis; F, female; GI, gastrointestinal; HR, heart rate; PE, pulmonary embolism; SD, standard deviation; UPE, unsuspected pulmonary embolism; VTE, venous thromboembolism.

Note: Bold *p* -values are statistically significant.

The rate of saddle PE was greater among patients with acute symptomatic PEs (21 out of 119; 18%) than in patients with UPE (15 out of 170; 9%), being this difference statistically significant ( *p* = 0.025).

Of note, a greater proportion of patients with saddle PE were diagnosed as having acute symptomatic PE (58 vs. 39%; *p* = 0.025) compared with those with nonsaddle PE.

Patients with saddle PE were more likely to have PE-related symptoms (69 vs. 51%; *p* = 0.042), tachycardia (44 vs. 24%; *p* = 0.01), and oxygen saturation <95% (56 vs. 31%; *p* = 0.004) compared with patients with nonsaddle PE.

Table 2 summarizes the main information regarding PE management and PE treatment with no differences according to saddle versus nonsaddle PE. Regarding the outcomes, notably the overall and PE-related mortality within 30 days were 15 and 2%, respectively, with no significant differences according to saddle versus nonsaddle PE. The overall rate of 30-day VTE recurrence (5%) and major bleeding (2%) were also found to be similar in both groups.

**Table 2:** Management of patients and 30-day outcomes according to the presence of saddle versus nonsaddle pulmonary embolism

	Overall ( *n* = 289) *n* (%)	Saddle PE ( *n* = 36) *n* (%)	Nonsaddle PE ( *n* = 253) *n* (%)	*p* -Value
Management setting				0.21
Inpatient at PE diagnosis	39 (14)	3 (8)	36 (14)	
Outpatient (<24 hours after diagnosis)	128 (44)	13 (36)	115 (46)	
Hospital admission in general ward	122 (42)	20 (56)	102 (40)	
Management				
Anticoagulation	289 (100)	36 (100)	253 (100)	>0.5
Fibrinolysis	5 (2)	1 (3)	4 (2)	0.49
Cava filter	12 (3)	3 (8)	9 (4)	0.18
Outcomes within 30 days				
Overall mortality	42 (15)	5 (14)	37 (15)	>0.5
PE-related mortality	5 (2)	2 (6)	3 (1)	0.12
Cancer-related mortality	34 (12)	3 (8)	31 (12)	>0.5
VTE recurrence	6 (2)	1 (3)	5 (2)	>0.5
Major bleeding	14 (5)	1 (3)	13 (5)	>0.5
In-hospital outcomes				
In-hospital overall mortality	23 (8)	4 (11)	19 (8)	>0.5
In-hospital PE related mortality	4 (1)	2 (6)	2 (1)	0.07
In-hospital cancer related mortality	19 (7)	2 (6)	17 (7)	>0.5
In-hospital VTE recurrence	5 (2)	1 (3)	4 (2)	>0.5
In-hospital major bleeding	6 (2)	0 (0)	6 (2)	>0.5

Abbreviations: PE, pulmonary embolism; VTE, venous thromboembolism.

### Radiological Findings of Saddle versus Nonsaddle Pulmonary Embolism

The radiological findings are shown in Table 3 . Of note, signs of RV overload including a RV/LV ratio ≥1 (22 vs. 4%; *p* < 0.001) and interventricular septum displacement (53 vs. 20%; *p* < 0.001) were more common in patients with saddle compared with nonsaddle PE.

**Table 3:** Radiological findings according to the presence of saddle versus non-saddle pulmonary embolism

	Overall ( *n* = 289) *n* (%)	Saddle PE ( *n* = 36) *n* (%)	Non-saddle PE ( *n* = 253) *n* (%)	*p* -Value
Radiological findings related to RV overload				
RV/LV ratio ≥1	18 (6)	8 (22)	10 (4)	**<0.001**
Septum displacement	69 (24)	19 (53)	50 (20)	**<0.001**
Suprahepatic vein reflux	38 (13)	8 (22)	30 (12)	0.11
At least one of the above	88 (30)	21 (58)	67 (27)	**<0.001**
Pulmonary arteries involved				**<0.001**
Central (main/lobar)	42 (15)	8 (22)	34 (13)	
Peripheral (segmentary/subsegmentary)	91 (31)	0 (0)	91 (36)	
Both central and peripheral	156 (54)	28 (78)	128 (51)	
Additional findings				
Lung nodules	113 (39)	11 (31)	102 (40)	0.26
Cancer progression a	74 (26)	9 (25)	65 (26)	>0.5
Carcinomatous lymphangitis	13 (4)	0 (0)	13 (5)	0.38
Pleural effusion	90 (31)	10 (28)	80 (32)	>0.5
Pericardial effusion	27 (9)	5 (11)	22 (9)	>0.5
Other radiological findings	148 (51)	18 (50)	130 (51)	>0.5

Abbreviations: LV, left ventricle; PE, pulmonary embolism; RV, right ventricle.

Note: Bold *p* -values are statistically significant.

a According to RECIST criteria. RECIST criteria are a set of radiological criteria that standardizes and simplifies treatment response criteria for neoplastic diseases and classifies them into 4 clearly defined categories: Complete response, Partial response, Progressive disease and Stable disease.

### Saddle Pulmonary Embolism According to an Acute Symptomatic versus Unsuspected Event

The main differences between patients with acute symptomatic saddle PE and patients diagnosed with unsuspected saddle PE are shown in Table 4 . Among the 36 patients with saddle PE, 15 (42%) were incidentally diagnosed. Patients with unsuspected saddle PE were more often receiving chemotherapy than those with acute symptomatic saddle PE (80 vs. 43%, respectively). Among patients with unsuspected saddle PE, 73% presented no symptoms on evaluation. Compared with unsuspected saddle PE, acute symptomatic saddle PE was associated with abnormal vital signs at diagnosis (5 vs. 0% with blood pressure <100 mm Hg, 71 vs. 7% with heart rate >100 bpm, and 33 vs. 0% with oxygen saturation below 90%, *p* > 0.5, <0.001. and 0.027, respectively), as well as septum displacement in the CT scan (71 vs. 27%, *p* = 0.008).

**Table 4:** Comparison of the main clinical features and outcomes of patients with cancer and saddle pulmonary embolism according to acute symptomatic versus unsuspected events

	Acute symptomatic saddle PE ( *n* = 21) Mean ± SD/ *n* (%)	Unsuspected saddle PE ( *n* = 15) Mean ± SD/ *n* (%)	*p* -Value
Age (y)	63.86 ± 10.9	67.33 ± 10.6	0.35
Gender (F)	8 (38)	6 (40)	>0.5
Previous VTE	8 (38)	3 (20)	0.3
Chronic heart condition	0 (0)	1 (7)	0.42
Chronic lung condition	6 (29)	1 (7)	0.2
Metastatic disease on presentation	17 (81)	10 (67)	0.44
Active chemotherapy treatment	9 (43)	12 (80)	**0.026**
Outpatient diagnosis	19 (91)	14 (93)	>0.5
Management setting			**<0.001**
Inpatient at PE diagnosis	2 (10)	1 (7)	
Outpatient (<24 hours after diagnosis)	0 (0)	13 (86)	
Hospital admission in general ward	19 (90)	1 (7)	
Symptoms			
None	0 (0)	11 (73)	**<0.001**
Dyspnea	20 (95)	4 (27)	**<0.001**
Chest pain	5 (24)	1 (7)	0.37
Syncope	4 (19)	0 (0)	0.13
Vital signs			
Arterial hypotension BP < 100 mm Hg	1 (5)	0 (0)	>0.5
Tachycardia HR > 100 bpm	15 (71)	1 (7)	**<0.001**
Oxygen saturation <90%	7 (33)	0 (0)	**0.027**
Oxygen saturation <95%	20 (95)	0 (0)	**<0.001**
Radiological findings related to RV overload			
RV/LV ratio ≥1	6 (29)	2 (13)	0.42
Septum displacement	15 (71)	4 (27)	**0.008**
Suprahepatic vein reflux	6 (29)	2 (13)	0.42
At least one of the above	16 (76)	5 (33)	**0.01**
Additional radiological findings			
Lung nodules	6 (29)	5 (33)	>0.5
Cancer progression a	6 (29)	3 (20)	>0.5
Carcinomatous lymphangitis	0 (0)	0 (0)	>0.5
Pleural effusion	7 (33)	3 (20)	0.47
Pericardial effusion	4 (19)	0 (0)	0.125
Other radiological findings	11 (52)	7 (47)	>0.5
Outcomes within 30 days			
Overall mortality	5 (24)	0 (0)	= 0.06
PE-related mortality	2 (10)	0 (0)	>0.5
Cancer-related mortality	3 (14)	0 (0)	0.25
VTE recurrence	1 (5)	0 (0)	>0.5
Major bleeding	1 (5)	0 (0)	>0.5

Abbreviations: BP, blood pressure; HR, heart rate; LV, left ventricle; PE, pulmonary embolism; RV, right ventricle; VTE, venous thromboembolism.

Note: Bold *p* -values are statistically significant.

a According to RECIST criteria. RECIST criteria are a set of radiological criteria that standardizes and simplifies treatment response criteria for neoplastic diseases and classifies them into 4 clearly defined categories: Complete response, Partial response, Progressive disease and Stable disease.

Only one patient (7%) with saddle UPE diagnosed in the outpatient setting required hospitalization while all the patients (100%) with acute symptomatic saddle PE were admitted to hospital ( *p* < 0.001). There was a trend toward a lower overall 30-day mortality in patients with unsuspected saddle PE compared with acute symptomatic PE (0 vs. 24%, *p* = 0.06).

## Discussion

We report a large cohort of consecutive patients with cancer-associated PE showing a notable prevalence of saddle PE of 12.5% in the overall cohort and 9% in those with UPE, taking into account that the imaging tests were specifically reviewed by a senior chest radiologist. Our results show a slightly higher prevalence of saddle PE than previously reported in two recent retrospective studies by Prentice et al 33 (10,660 hospital admissions, 49% metastatic cancer, and saddle PE prevalence of 4.5%) and by Banala et al 34 (193 episodes of UPE, 75% metastatic cancer, and saddle PE prevalence of 3.6%).

We hypothesize that these differences might be explained by the fact that a large proportion (82%) of our patients had metastatic cancer, representing a larger proportion of patients with advanced disease compared with the aforementioned studies. 31 32 However, our data do not support this theory, as the association of metastatic cancer and saddle PE was not significant and the proportion of patients with metastatic cancer was actually lower in saddle PE than nonsaddle PE (75 vs. 83%). Additionally, differences in the prevalence of saddle PE among studies might be related to the fact that the two previous real-world studies 33 34 did not include a thorough radiological review of CT scans, and thus saddle PE could have been underreported. In this regard, another retrospective study by Kwak et al, 35 in the general population, reported a prevalence of 9.1% of saddle PE after specific radiological review similar to what was done in our study.

In our dataset, patients with saddle PE were more likely to be symptomatic and present with altered vital signs at PE diagnosis compared with patients with nonsaddle PE. Interestingly, 31% of patients with saddle PE were asymptomatic. However, in view of the lack of studies in this regard, comparison with other studies could not be made, since Prentice et al 33 provided no data on either symptoms or vital signs and Banala et al 34 did not specifically analyzed saddle PE. Nonetheless, we believe that this is an original finding in patients with cancer that emphasizes the clinical-radiological dissociation previously described in isolated case reports. 36 37

Notably, patients with saddle PE in our study more frequently had signs of RV overload compared with nonsaddle PE, in line with the study by Kwak et al. 35 In contrast, in a retrospective study of 52 patients with cancer and central acute symptomatic PE with thorough revision of CT scans, Yusuf et al 38 did not find differences in signs of RV overload according to saddle versus nonsaddle central PE.

Despite having found significant differences in PE presentation and signs of RV overload in our study, we could not demonstrate a worse overall or PE-related 30-day mortality according to saddle versus nonsaddle PE. Up to now, only Prentice et al 33 and Yusuf et al 38 have published data regarding the prognostic value of saddle PE in patients with cancer, albeit with conflicting results.

Similarly to our results, many of the studies regarding saddle PE in the general population found no difference in overall mortality between saddle and nonsaddle PE at 30 days, 35 39 40 41 42 although data from other heterogenous uncontrolled observational studies 43 44 suggest a greater risk of in-hospital mortality in patients with saddle PE. Taking all of this into account, the prognostic impact of saddle PE remains inconclusive.

Unsuspected PE has shifted the paradigm of PE in patients with cancer, but very little information is available regarding unsuspected saddle PE and short-term outcomes. To the best of our knowledge, no other study has compared unsuspected saddle PE with acute symptomatic saddle PE. In our series, saddle PE was diagnosed as an incidental finding in a high proportion of patients (42%). This proportion exceeded our initial expectations and, we believe, has not been previously reported, suggesting that unsuspected saddle PE is a common presentation of VTE in patients with cancer.

From an epidemiological point of view (baseline characteristics), it is noteworthy patients with unsuspected saddle PE were more frequently receiving active chemotherapy treatment compared with patients with acute saddle PE. This difference might be influenced by this group of patients more frequently undergoing scheduled CT studies with the subsequent identification of unsuspected VTE events.

Related to clinical presentation and outcomes, it is remarkable that patients with unsuspected saddle PE were often asymptomatic (73%) and rarely required hospital admission (7%). Septum displacement was consistently less frequent in patients with unsuspected saddle PE compared with those with acute symptomatic saddle PE, although no differences in other variables related to RV overload were found. Taking into account, the absence of significant differences in 30-day outcomes in patients with unsuspected saddle PE compared with acute saddle PE, it is further underscored that patients with saddle PE presented a high degree of clinical-radiological dissociation, and that clinical signs may be better predictors of short-term major adverse outcomes.

## Strengths and Limitations

While from a clinical point of view, the present study has several strengths that have been indicated above, there are also several limitations. Our study was conducted in a single center, retrospective, and not randomized/interventional in nature. Even though patients were consecutively included, the retrospective nature of the study and patient recruitment from the Medical Oncology Department (and not every PE diagnosed in the Radiology Department) are potential sources of selection bias. It is of note that the differences found in signs of RV overload on CT may be affected by the fact that conventional CT scans used in 59% of the patients, without pulmonary angiography, cannot usually identify these signs. Moreover, the assessment of clinical outcomes among patients with and without saddle PE lacks precision due to low numbers of the outcomes of interest. In addition, RV overload with transthoracic echocardiogram and cardiac biomarkers were seldom studied in our cohort because they were not routinely measured during the study period. This precluded the adjustment of 30-day outcomes with the current guideline-recommended risk-stratification tools. 16 45 In this regard, it would be interesting to design specific prospective multicentric studies addressed to overcome this particular drawback to determine the prognostic impact of saddle PE more precisely.

Sample size was a limitation to find more conclusive results regarding short-term prognostic impact of saddle PE in patients with cancer. Further prospective studies are needed to assess the safety of outpatient management of cancer-related PE.

## Conclusion

In conclusion, the present study sheds light on the prevalence, clinical characteristics, and outcomes of patients with cancer and saddle PE versus nonsaddle PE. Saddle PE was found in a relevant proportion of patients, including those with UPE.
